# Mitochondrial blood‐based biomarker is related to fitness and aging in a sex‐dependent manner

**DOI:** 10.1002/alz70855_106196

**Published:** 2025-12-24

**Authors:** Riley E Kemna, Amanda Szabo‐Reed, Paul J Kueck, Jenae Pennington, Hana Mayfield, Casey S. John, Eric D Vidoni, Jill K Morris

**Affiliations:** ^1^ University of Kansas Alzheimer's Disease Research Center, Fairway, KS, USA; ^2^ University of Kansas Medical Center, Kansas City, KS, USA; ^3^ University of Kansas Alzheimer's Disease Research Center, Kansas City, KS, USA; ^4^ University of Kansas Medical Center, Alzheimer's Disease Research Center, Fairway, KS, USA

## Abstract

**Background:**

A sedentary lifestyle increases risk for Alzheimer's Disease (AD), whereas exercise has been shown to benefit brain health. Other physiological factors, such as female sex is linked to lower physical fitness and can increase risk of AD, which might impact exercise benefit to the brain. Exploring cellular mechanisms that underly fitness in older adults is essential to understanding the relationship between exercise and AD risk, and how sex might impact this interaction.

**Methods:**

We collected blood from 34 cognitively healthy older adults (age 65+, 18 male, 16 female) who were enrolled in the COMbined Exercise Trial (COMET; NCT04848038) at the University of Kansas ADRC. Subjects underwent a blood draw and clinical assessments for physical fitness (graded exercise test) and body composition (dual x‐ray absorptiometry) for cross‐sectional analysis. Blood was collected in ACD tubes and lymphocytes were isolated. Fluorescent stains used were MitoTracker, Annexin V, MitoSOX, TMRE, and Hoechst and analyzed by flow cytometry (Cytek Aurora). Flow cytometer outcomes were used to calculate a composite mitochondrial health index (MHI).

**Results:**

Our cohort had a mean age of 70.9 [3.5] years and included 32.3% *APOE4* carriers. As expected, males had higher lean mass and VO_2peak_ than females (*p* = 0.001 and 0.014), but groups did not differ in body mass index (*p* = 0.506). Males had a higher MHI compared to females (*p* = 0.014). Within each sex, we observed unique metabolic relationships. In males, there was an age‐associated decline in MHI (β= ‐0.618, *p* = 0.008). In females, our systemic measure of mitochondrial reactive oxygen species (ROS) had a negative relationship with lean mass (β= ‐0.805, *p* <0.001) and oxygen uptake efficiency slope (OUES) (β= ‐0.520, *p* = 0.039).

**Conclusion:**

We combined a MHI with measures related to physical fitness in a cognitively healthy older adult population. We explored physiological factors that impact fitness and response to exercise training, such as sex. We observed relationships between mitochondrial measures and OUES and lean mass in females, whereas males had higher MHI overall. Sex‐depended differences in mitochondrial health and ROS likely contribute to the variable response to exercise training between males and females, and might help explain differences in AD risk between sex.

